# Decreased pain at 12 weeks post-operatively is associated with superior shoulder range of motion after anatomic and reverse total shoulder arthroplasty and is sustained up to two years

**DOI:** 10.1016/j.jseint.2026.101662

**Published:** 2026-02-11

**Authors:** Robert J. Cueto, Kevin A. Hao, Logan Wright, Keegan M. Hones, Jonathan O. Wright, Thomas W. Wright, Kevin W. Farmer, Tyler J. LaMonica, Bradley S. Schoch, Joseph J. King

**Affiliations:** a Department of Orthopaedic Surgery & Sports Medicine, University of Florida, Gainesville, FL, USA; b College of Medicine, University of Florida, Gainesville, FL, USA; c Department of Orthopaedic Surgery, Mayo Clinic, Jacksonville, FL, USA

**Keywords:** Pain, Total shoulder arthroplasty, Anatomic shoulder arthroplasty, Reverse shoulder arthroplasty, Post-operative pain, Range of motion

## Abstract

**Background:**

While immediate post-operative pain control after total shoulder arthroplasty (TSA) has been associated with improved long-term functional outcomes, the effect of pain levels beyond the initial recovery period remains unclear. This study aimed to evaluate whether decreased pain at 12 weeks post-operatively after anatomic TSA (aTSA) and reverse TSA (rTSA) is associated with improved shoulder range of motion (ROM) and pain control up to 2 years post-operatively compared to patients with elevated pain during the post-operative recovery period.

**Methods:**

We performed a retrospective review of 605 primary TSAs (206 aTSA, 399 rTSA) at a single institution from 2007 to 2022. Perioperatively, patients received a cervical paravertebral nerve catheter. Post-operatively, patients were discharged on a multimodal oral analgesic regimen consisting of both opioid and nonopioid medications. Pre-operative to post-operative improvement in ROM was evaluated at 6 weeks, 12 weeks, 6 months, 1 year, and 2 years follow-up. Mixed-effects models were used to evaluate whether minimal post-operative pain (defined as patient-reported pain at worst < 3/10) at 12-week follow-up was associated with improved ROM (forward elevation [FE], abduction, external rotation, and internal rotation [IR]) and pain at each of the next follow-up time point compared to those with elevated pain. Clinical significance was evaluated by comparing improvement to the minimum clinically important difference (MCID) and substantial clinical benefit (SCB).

**Results:**

For aTSA, patients with pain < 3/10 at 12 weeks demonstrated significantly greater pre-operative to post-operative improvement in abduction, FE, and IR at all follow-up points, including 2 years post-operatively. For rTSA, patients with minimal pain at 12 weeks had significantly greater pre-operative to post-operative improvement in abduction and IR at all follow-up points, including 2 years post-operatively. For both aTSA and rTSA cohorts, patients with minimal pain at 12 weeks had significantly greater mean improvement in daily pain and pain at worst at all follow-up points, including 2 years post-operatively. Patients with minimal pain after aTSA achieved the MCID earlier for abduction and FE and the SCB earlier for abduction, FE, and IR. Patients with minimal early pain after rTSA achieved the MCID earlier for FE and the SCB earlier for external rotation and IR.

**Conclusion:**

Minimal pain at 12 weeks post-operatively is associated with sustained superior ROM gains and reduced pain up to 2 years post-operatively after aTSA and rTSA. Future prospective studies are needed to determine what patient and surgery factors lead to poor early pain control and which can be optimized to improve the functional benefits of TSA.

Pain during the post-operative recovery period following total shoulder arthroplasty (TSA), including both anatomic (aTSA) and reverse (rTSA) procedures, remains a significant clinical challenge.^14^^,^^19^ While the majority of patients undergoing TSA experience pain relief compared to pre-operative levels, up to 15%-33% may continue to report shoulder pain equal to or greater than baseline at 12 weeks post-operatively, with some experiencing disabling symptoms.^19^^,^^20^ Although numerous studies have examined various strategies to mitigate post-operative pain after TSA, relatively little is known about the impact of post-operative pain during the recovery period on long-term outcomes.^22^^,^^23^ Furthermore, as post-operative rehabilitation allows patients to gradually progress post-operative range of motion (ROM) and shoulder strength after TSA, excessive post-operative pain can prohibit adequate participation in therapy as well as compliance with home exercises, possibly leading to inferior shoulder function ultimately.^6^^,^^16^

This study aimed to evaluate whether lower patient-reported pain at 12 weeks is associated with superior pre-operative to post-operative improvement in ROM up to 2 years post-operatively following aTSA and rTSA. Secondarily, we evaluated whether patients with lower pain at 12 weeks had sustained superior pain relief up to 2 years post-operatively. We hypothesized that minimal post-operative pain during the recovery period would be associated with greater pre-operative to post-operative improvement in shoulder ROM and improved pain relief after TSA with sustained benefits up to 2 years post-operatively.

## Materials and methods

Following institutional review board approval, we retrospectively reviewed a prospectively collected shoulder arthroplasty database from a single academic center. All primary TSAs performed in adult patients between 2007 and 2022 with a clinic visit pre-operatively, at 12-week follow-up, and at 2-year follow-up were included. While follow-up at 12 weeks and 2 years was required to be included in this study, all available follow-up visits (6 weeks, 12 weeks, 6 months, 1 year, and 2 years) were included for each patient when available. Patients undergoing revision procedures or with pre-operative diagnoses of infection, acute fracture, fracture sequelae, tumor, or nerve injury were excluded. To create a more homogenous cohort, only patients who had a pre-operative diagnosis of primary osteoarthritis, rotator cuff tear, or rotator cuff arthropathy were included. In total, 206 aTSAs in 191 patients and 399 rTSAs in 364 patients were included for analysis.

### Surgical intervention and rehabilitation

All surgeries were performed by one of 5 fellowship-trained shoulder surgeons using implants from multiple manufacturers. A standard deltopectoral approach was used in all cases. The decision to repair the subscapularis in the setting of rTSA was left to the discretion of the treating surgeon and was often based on tissue quality, tendon excursion intraoperatively, and perceived muscle quality based on pre-operative imaging. Post-operatively, all patients were instructed to complete a standard rehabilitation protocol consisting of a physical therapist–directed home exercise program. A sling was worn for 2 to 6 weeks post-operatively. Pendulum exercises and ROM limited to passive forward elevation (FE) and active external elevation (ER) to neutral were permitted starting at three weeks based on surgeon recommendation. Active ROM without limitations was initiated at 6 weeks, followed by strengthening exercises at 12 weeks.

### Pain management

Perioperatively, the standard of care at our institution involves patients receiving a cervical paravertebral catheter placed 1-2 hours prior to surgery. Paravertebral infusions consisted of 0.2% ropivacaine at 10 mL per hour, with a 10 mL bolus available following 60-min lockout periods. Infusions were typically maintained for 2 to four days after surgery. Patients also had access to as-needed oral opioid and nonopioid analgesics to augment the effect of continuous nerve catheters. Intravenous medications such as morphine and hydromorphone were used for severe pain as needed if the patient was admitted post-operatively. Commonly used oral analgesics included acetaminophen, nonsteroidal anti-inflammatory drugs, and oxycodone. If admitted, patients were seen twice daily by the inpatient acute pain service following surgery. Upon discharge, patients were contacted at least once daily by telephone. Catheter removal was performed by a patient-designated assistant during phone consultation with the acute pain service following infusion pause and confirmation of complete return of normal motor and sensory function.

### Range of motion assessment

Shoulder ROM was assessed pre-operatively and at all follow-up visits by a trained research assistant or a physical therapist using a goniometer following a standardized protocol. Measurements included active abduction, FE, and ER. Active internal rotation (IR) was assessed using the most cephalad vertebral level reached by the thumb behind the patient's back and scored according to the Flurin scale: 0 = none, 1 = hip, 2 = buttocks, 3 = sacrum, 4 = L5-L4, 5 = L3-L1, 6 = T12-T8, 7 = T7 or higher.

### Pain assessment

**Figure 1:**
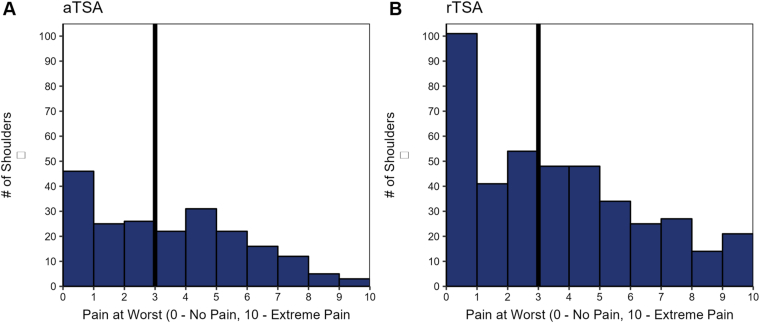
Histogram of pain at worst at 12-week follow-up for aTSA (**A**) and rTSA (**B**). The vertically oriented bolded black line indicates the cutoff point that was chosen to segment the minimal pain control cohort (ie, pain at worst < 3) vs. the uncontrolled pain cohort (pain at worst ≥ 3). *aTSA*, anatomic total shoulder arthroplasty; *rTSA*, reverse total shoulder arthroplasty.

Patients were asked to report their pain on a daily basis and pain at worst pre-operatively and at each post-operative visit. Pain was assessed using a visual analog scale, with 0 representing no pain and 10 representing extreme pain. Based on 12-week pain at its worst score, patients were stratified into 2 cohorts: pain < 3 (minimal pain) or pain ≥ 3 (elevated pain) (Fig. 1).

### Missing data

To reduce the influence of missing data on generated estimates, we elected to utilize multivariate random forest imputation.^13^ This method is commonly used in the shoulder literature.^8^^,^^10^^,^^11^^,^^27^ First, missing values are imputed with a rough estimate. Subsequently, a predictive random forest is trained to reimpute missing values iteratively until convergence. The predictive random forest model was trained using demographic information, pre-operative and post-operative ROM, and pain scores available from clinical follow-up visits. Random forest imputation was performed using the *simputation* package.^26^

### Statistical analysis

We divided aTSA and rTSA cohorts based upon whether they reported pain at worst to be < 3 vs. ≥ 3 on a ten-point scale. This threshold was chosen based on a visual analysis of the distribution of patient-reported pain at worst at 12 weeks post-operatively in our cohort (Fig. 1), as well as by a prior report by Hao et al^9^ that found the patient acceptable symptomatic state threshold for pain at worst to be 3 out of 10.

Demographics of included aTSAs and rTSAs were characterized descriptively by pain group. To account for variable data availability depending on whether patients followed up at all post-operative clinic visits (6 weeks, 12 weeks, 6 months, 1 year, 2 years), we utilized mixed-effect models with patients designated as random effects to compare pre-operative to post-operative improvement in ROM and pain scores based on whether patient-reported pain at worst at 12-week follow-up was < 3 or ≥ 3. Linear mixed-effects models were generated using the *lme4* package.^2^ The minimum clinically important difference (MCID) and substantial clinical benefit (SCB) thresholds defined by Simovitch et al^25^ in patients with osteoarthritis and corresponding procedure (aTSA or rTSA) were utilized to evaluate for clinical significance. All statistical analyses were performed in R Software (version 4.2.0, R Core Team, Vienna, Austria) using an α of 0.05.

## Results

### Anatomic total shoulder arthroplasty

Of the 206 aTSAs included, 70 patients (34%) were classified as having minimal pain and 136 (66%) were classified as having elevated pain by 12 weeks post-operatively. Baseline demographics and comorbidities were similar between groups (Table I).

**Table I:** Comparison of demographics of included shoulders undergoing aTSA and rTSA with minimal and elevated post-operative recovery period pain at 12 weeks post-operatively.

Characteristic	aTSA (n = 206)			rTSA (n = 399)		
	Minimal pain (n = 70)	Elevated pain (n = 136)	*P* value	Minimal pain (n = 138)	Elevated pain (n = 261)	*P* value
Age at surgery (yr)	66.6 ± 7.5	65.7 ± 7.2	.391	72.2 ± 6.6	70.4 ± 8.4	**.016**
BMI (kg/m^2^)	30.5 ± 7.6	30.9 ± 6.8	.669	29.4 ± 5.5	29.3 ± 6.5	.894
Female sex	38.6% (27)	42.6% (58)	.654	50.0% (69)	56.3% (147)	.246
Previous surgery	10.0% (7)	16.2% (22)	.292	34.1% (47)	35.2% (92)	.826
Comorbidities						
Inflammatory arthritis	11.4% (8)	5.1% (7)	.154	4.3% (6)	6.1% (16)	.645
Hypertension	50.0% (35)	60.3% (82)	.182	62.3% (86)	62.8% (164)	.914
Heart disease	10.0% (7)	11.8% (16)	.818	18.8% (26)	16.1% (42)	.487
Diabetes	12.9% (9)	15.4% (21)	.681	11.6% (16)	22.2% (58)	**.010**
Tobacco use	4.3% (3)	4.4% (6)	1.000	2.2% (3)	4.2% (11)	.396
Chronic renal failure	2.9% (2)	0.7% (1)	.267	0.7% (1)	3.1% (8)	.172
Chronic liver failure	0.0% (0)	1.5% (2)	.549	0.0% (0)	0.0% (0)	1.000
Preoperative diagnosis			.340			.662
OA with an intact rotator cuff	98.6% (69)	100.0% (136)	-	33.3% (46)	26.1% (68)	-
OA with a torn rotator cuff	0.0% (0)	0.0% (0)	-	17.4% (24)	16.9% (44)	-
Rotator cuff arthropathy	0.0% (0)	0.0% (0)	-	40.6% (56)	48.7% (127)	-
Rotator cuff tear	0.0% (0)	0.0% (0)	-	6.5% (9)	6.1% (16)	-
Rheumatoid arthritis	1.4% (1)	0.0% (0)	-	1.4% (2)	1.5% (4)	-
Avascular necrosis	0.0% (0)	0.0% (0)	-	0.7% (1)	0.8% (2)	-
Tranexamic acid given intraoperatively	30.0% (21)	25.7% (35)	.514	42.0% (58)	42.9% (112)	.962

*aTSA*, anatomic total shoulder arthroplasty; *rTSA*, reverse total shoulder arthroplasty; *BMI*, body mass index; *OA*, osteoarthritis.

Bold values indicate *P* < .05.

**Figure 2:**
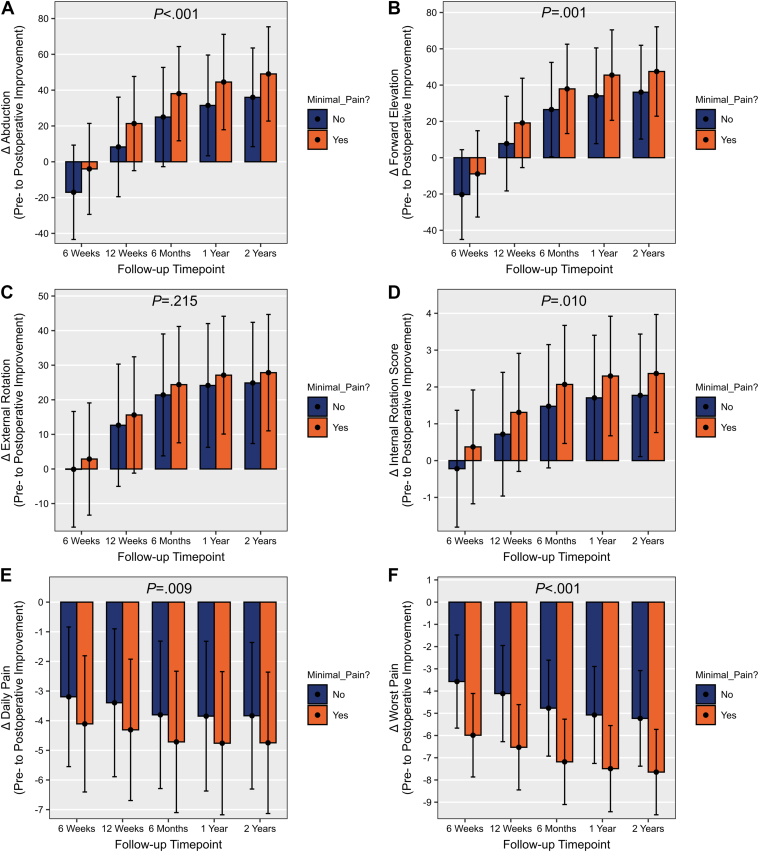
Pre-operative to post-operative improvement after aTSA in abduction (**A**), forward elevation (**B**), external rotation (**C**), internal rotation score (**D**), daily pain (**E**), and worst pain (**F**). *aTSA*, anatomic total shoulder arthroplasty.

Patients with minimal pain during the post-operative recovery period demonstrated significantly greater pre-operative to post-operative improvement in abduction (*P* < .001), FE (*P* = .001), and IR (*P* = .010) compared to those with elevated pain up to 2 years post-operatively. However, pre-operative to post-operative improvement of ER was not significantly different between those with minimal vs. elevated pain up to 2 years post-operatively (*P* = .215) (Fig. 2 and Table II). In patients with minimal pain during the post-operative recovery period, the MCID was achieved earlier for abduction and FE (12 weeks vs. 6 months for both), while time to achieve the MCID for ER and IR did not differ between groups (12 weeks post-operatively for both). Furthermore, patients with minimal pain during the post-operative recovery period achieved the SCB earlier for abduction, FE, and IR (6 months vs. 1 year; 6 months vs. 1 year; and 12 weeks vs. 6 months post-operatively, respectively). Time to achieve the SCB did not differ between groups for ER (6 months post-operatively for both).

**Table II:** Comparison of range of motion and pain scores derived from mixed-effect models between patients undergoing anatomic total shoulder arthroplasty with minimum and elevated pain during the post-operative recovery period.

ROM measure	Minimal pain	Elevated pain	*P* value
Abduction			**<.001**
Pre-operative to 6 weeks	−4.0 ± 25.3 [−10.2 to 2.3]	−17.0 ± 26.3 [−21.7 to −12.3]	-
Pre-operative to 12 weeks	21.4 ± 26.2 [15.2-27.6]	8.3 ± 27.7 [3.7-13.0]	-
Pre-operative to 6 mo	38.1 ± 26.2 [31.9-44.2]	25.0 ± 27.6 [20.4-29.7]	-
Pre-operative to 1 yr	44.5 ± 26.5 [38.3-50.6]	31.4 ± 28.0 [26.8-36.1]	-
Pre-operative to 2 yr	48.9 ± 26.2 [42.7-55.0]	35.8 ± 27.5 [31.2-40.5]	-
Forward elevation			**.001**
Pre-operative to 6 weeks	−8.9 ± 23.8 [−14.8 to −3.1]	−20.2 ± 24.8 [−24.7 to −15.8]	-
Pre-operative to 12 weeks	19.1 ± 24.6 [13.3-24.9]	7.8 ± 26.1 [3.4-12.2]	-
Pre-operative to 6 mo	37.9 ± 24.6 [32.1-43.7]	26.6 ± 26.0 [22.2-30.9]	-
Pre-operative to 1 yr	45.4 ± 24.9 [39.6-51.2]	34.1 ± 26.4 [29.7-38.4]	-
Pre-operative to 2 yr	47.3 ± 24.6 [41.5-53.1]	36.0 ± 25.9 [31.6-40.4]	-
External rotation			.215
Pre-operative to 6 weeks	2.8 ± 16.2 [−1.1 to 6.8]	−0.1 ± 16.7 [−3.1 to 2.9]	-
Pre-operative to 12 weeks	15.6 ± 16.8 [11.7-19.6]	12.7 ± 17.7 [9.7-15.7]	-
Pre-operative to 6 mo	24.4 ± 16.8 [20.4-28.3]	21.5 ± 17.6 [18.5-24.4]	-
Pre-operative to 1 yr	27.0 ± 17.0 [23.1-31.0]	24.1 ± 17.9 [21.2-27.1]	-
Pre-operative to 2 yr	27.8 ± 16.8 [23.8-31.7]	24.8 ± 17.5 [21.9-27.8]	-
Internal rotation score			**.010**
Pre-operative to 6 weeks	0.4 ± 1.5 [0.0-0.8]	−0.2 ± 1.6 [−0.5 to 0.1]	-
Pre-operative to 12 weeks	1.3 ± 1.6 [0.9-1.7]	0.7 ± 1.7 [0.4-1.0]	-
Pre-operative to 6 mo	2.1 ± 1.6 [1.7-2.4]	1.5 ± 1.7 [1.2-1.8]	-
Pre-operative to 1 yr	2.3 ± 1.6 [1.9-2.7]	1.7 ± 1.7 [1.4-2.0]	-
Pre-operative to 2 yr	2.4 ± 1.6 [2.0-2.7]	1.8 ± 1.7 [1.5-2.1]	-
Pain on a daily basis			**.009**
Pre-operative to 6 weeks	−4.1 ± 2.3 [−4.7 to −3.5]	−3.2 ± 2.4 [−3.6 to −2.8]	-
Pre-operative to 12 weeks	−4.3 ± 2.4 [−4.8 to −3.7]	−3.4 ± 2.5 [−3.8 to −3.0]	-
Pre-operative to 6 mo	−4.7 ± 2.4 [−5.2 to −4.1]	−3.8 ± 2.5 [−4.2 to −3.4]	-
Pre-operative to 1 yr	−4.7 ± 2.4 [−5.3 to −4.1]	−3.8 ± 2.5 [−4.2 to −3.4]	-
Pre-operative to 2 yr	−4.7 ± 2.4 [−5.3 to −4.1]	−3.8 ± 2.5 [−4.2 to −3.4]	-
Pain at worst			**<.001**
Pre-operative to 6 weeks	−5.9 ± 1.9 [−6.3 to −5.4]	−3.5 ± 2.1 [−3.9 to −3.1]	-
Pre-operative to 12 weeks	−6.5 ± 1.9 [−6.9 to −6.0]	−4.1 ± 2.2 [−4.5 to −3.8]	-
Pre-operative to 6 mo	−7.1 ± 1.9 [−7.6 to −6.7]	−4.8 ± 2.2 [−5.1 to −4.4]	-
Pre-operative to 1 yr	−7.4 ± 1.9 [−7.9 to −7.0]	−5.1 ± 2.2 [−5.4 to −4.7]	-
Pre-operative to 2 yr	−7.6 ± 1.9 [−8.0 to −7.1]	−5.2 ± 2.2 [−5.6 to −4.9]	-

*ROM*, range of motion.

Minimal pain, defined as pain at worst < 3/10 at 12-week follow-up.

Values represent mean ± standard deviation [95% confidence interval] unless otherwise noted.

Bold indicates statistical significance.

In addition, patients in the minimal pain group reported significantly greater improvements in daily pain (*P* = .009) and pain at worst (*P* < .001) throughout follow-up compared to patients with elevated pain (Fig. 2 and Table II). The time to achieve the MCID and SCB did not differ between groups to achieve improvement in pain at worst (6 weeks post-operatively for the MCID and SCB for both groups).

### Reverse total shoulder arthroplasty

Of the 399 rTSAs, 138 patients (35%) were classified as having minimal pain during the post-operative recovery period and 261 (65%) were classified as having elevated pain by 12 weeks post-operatively. Patients in the minimal pain cohort were older (72 ± 7 vs. 70 ± 8 years, *P* = .016) and had a lower prevalence of diabetes (12% vs. 22%, *P* = .010) compared to those with elevated pain. Other demographics and comorbidities were similar (Table I).

**Figure 3:**
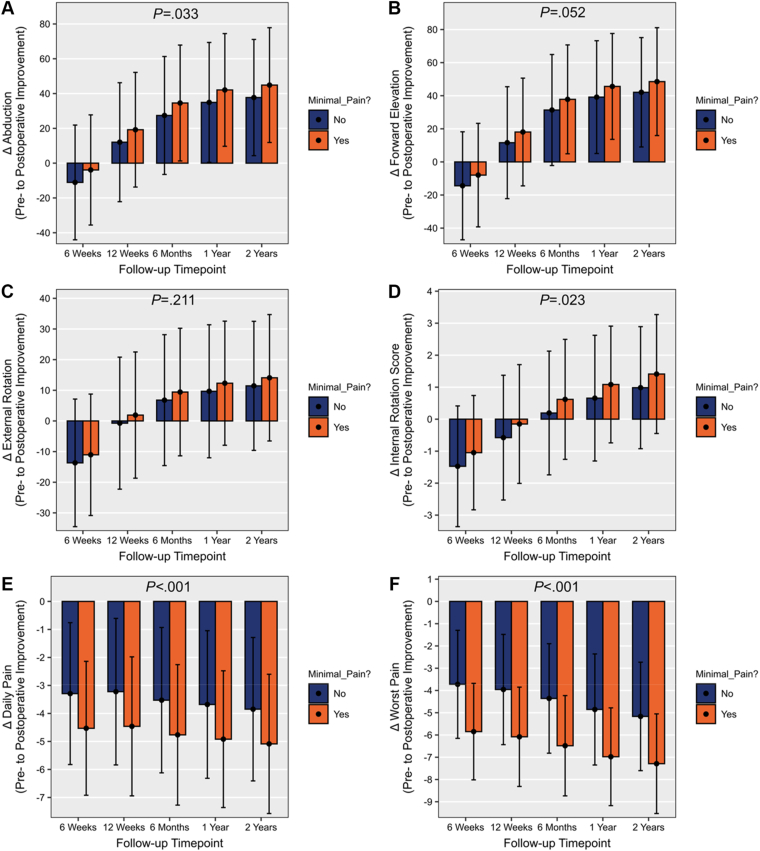
Pre-operative to post-operative improvement after rTSA in abduction (**A**), forward elevation (**B**), external rotation score (**D**), daily pain (**E**), and worst pain (**F**). *rTSA*, reverse total shoulder arthroplasty.

Patients with minimal pain during the post-operative recovery period demonstrated significantly greater pre-operative to post-operative improvement in abduction (*P* = .033) and IR (*P* = .023) compared to those with elevated pain up to 2 years post-operatively. However, pre-operative to post-operative improvement of FE and ER was not significantly different between those with minimal vs. elevated pain up to 2 years post-operatively (*P* = .052 and *P* = .211, respectively) (Fig. 3 and Table III). In patients with minimal pain during the post-operative recovery period, the MCID was achieved earlier for FE (12 weeks vs. 6 months post-operatively), while time to achieve the MCID in abduction, ER, and IR did not differ between groups (12 weeks, 6 months, and 6 months in both groups post-operatively, respectively). Furthermore, patients with minimal pain achieved the SCB earlier for ER and IR (6 months vs. 1 year and 1 year vs. 2 years post-operatively, respectively). Time to achieve the SCB did not differ between groups for abduction and FE (6 months for both).

**Table III:** Comparison of range of motion and pain scores derived from mixed-effect models between patients undergoing reverse total shoulder arthroplasty with minimal and elevated pain during the post-operative recovery period.

ROM measure	Minimal pain	Elevated pain	*P* value
Abduction			**.033**
Pre-operative to 6 weeks	−4.0 ± 31.7 [−9.5 to 1.5]	−11.1 ± 33.0 [−15.2 to −7.0]	-
Pre-operativeto 12 weeks	19.2 ± 33.0 [13.8-24.7]	12.1 ± 34.2 [8.0-16.2]	-
Pre-operative to 6 mo	34.5 ± 33.3 [29.1-40.0]	27.4 ± 33.9 [23.3-31.5]	-
Pre-operative to 1 yr	42.1 ± 32.4 [36.6-47.5]	35.0 ± 34.5 [30.9-39.0]	-
Pre-operative to 2 yr	44.8 ± 33.0 [39.3-50.2]	37.6 ± 33.4 [33.6-41.7]	-
Forward elevation			.052
Pre-operative to 6 weeks	−8.1 ± 31.3 [−13.5 to −2.6]	−14.4 ± 32.6 [−18.5 to −10.4]	-
Pre-operative to 12 weeks	18.1 ± 32.6 [12.7-23.5]	11.7 ± 33.8 [7.7-15.7]	-
Pre-operative to 6 mo	37.7 ± 32.9 [32.4-43.1]	31.4 ± 33.5 [27.3-35.4]	-
Pre-operative to 1 yr	45.6 ± 32.0 [40.2-51.0]	39.2 ± 34.1 [35.2-43.3]	-
Pre-operative to 2 yr	48.3 ± 32.6 [42.9-53.7]	42.0 ± 33.0 [37.9-46.0]	-
External rotation			.211
Pre-operative to 6 weeks	−11.1 ± 19.8 [−14.5 to −7.6]	−13.6 ± 20.8 [−16.2 to −11.0]	-
Pre-operative to 12 weeks	1.8 ± 20.6 [−1.6 to 5.3]	−0.7 ± 21.5 [−3.3 to 1.8]	-
Pre-operative to 6 mo	9.3 ± 20.8 [5.9-12.8]	6.8 ± 21.4 [4.2-9.3]	-
Pre-operative to 1 yr	12.3 ± 20.3 [8.9-15.7]	9.7 ± 21.7 [7.1-12.3]	-
Pre-operative to 2 yr	14.0 ± 20.6 [10.5-17.4]	11.4 ± 21.1 [8.8-14.0]	-
Internal rotation score			**.023**
Pre-operative to 6 weeks	−1.1 ± 1.8 [−1.4 to −0.7]	−1.5 ± 1.9 [−1.7 to −1.2]	-
Pre-operative to 12 weeks	−0.2 ± 1.9 [−0.5 to 0.1]	−0.6 ± 1.9 [−0.8 to −0.3]	-
Pre-operative to 6 mo	0.6 ± 1.9 [0.3-0.9]	0.2 ± 1.9 [0.0-0.4]	-
Pre-operative to 1 yr	1.1 ± 1.8 [0.8-1.4]	0.7 ± 2.0 [0.4-0.9]	-
Pre-operative to 2 yr	1.4 ± 1.9 [1.1-1.7]	1.0 ± 1.9 [0.7-1.2]	-
Pain on a daily basis			**<.001**
Pre-operative to 6 weeks	−4.4 ± 2.4 [−4.8 to −4.0]	−3.2 ± 2.5 [−3.5 to −2.8]	-
Pre-operative to 12 weeks	−4.4 ± 2.5 [−4.8 to −4.0]	−3.2 ± 2.6 [−3.5 to −2.9]	-
Pre-operative to 6 mo	−4.7 ± 2.5 [−5.1 to −4.3]	−3.5 ± 2.6 [−3.8 to −3.2]	-
Pre-operative to 1 yr	−4.9 ± 2.5 [−5.3 to −4.5]	−3.6 ± 2.6 [−3.9 to −3.3]	-
Pre-operative to 2 yr	−5.0 ± 2.5 [−5.5 to −4.6]	−3.8 ± 2.6 [−4.1 to −3.5]	-
Pain at worst			**<.001**
Pre-operative to 6 weeks	−5.6 ± 2.2 [−6.0 to −5.3]	−3.5 ± 2.4 [−3.8 to −3.2]	-
Pre-operative to 12 weeks	−6.1 ± 2.2 [−6.4 to −5.7]	−3.9 ± 2.5 [−4.2 to −3.6]	-
Pre-operative to 6 mo	−6.4 ± 2.2 [−6.8 to −6.1]	−4.3 ± 2.5 [−4.6 to −4.0]	-
Pre-operative to 1 yr	−6.9 ± 2.2 [−7.3 to −6.6]	−4.8 ± 2.5 [−5.1 to −4.5]	-
Pre-operative to 2 yr	−7.3 ± 2.2 [−7.6 to −6.9]	−5.1 ± 2.4 [−5.4 to −4.9]	-

*ROM*, range of motion.

Minimal pain, defined as pain at worst < 3/10 at 12-week follow-up.

Values represent mean ± standard deviation [95% confidence interval] unless otherwise noted.

Bold indicates statistical significance.

In addition, patients in the minimal pain group reported significantly greater improvements in daily pain (*P* < .001) and pain at worst (*P* < .001) throughout follow-up compared to patients with elevated pain (Fig. 3 and Table III). The time to achieve the MCID and SCB did not differ between groups for improvement in pain at worst (6 weeks post-operatively).

## Discussion

Despite advances in technique and implant design, achieving adequate and consistent long-term outcomes after TSA remains challenging.^1^^,^^17^^,^^21^ Prior literature has suggested that inadequate immediate post-operative pain control may negatively impact long-term functional outcomes following TSA.^14^ However, there remains a paucity of data investigating the relationship between pain levels beyond the immediate post-operative period and long-term outcomes. In this retrospective analysis, we found that patients with minimal pain (visual analog scale pain < 3/10) at early clinical follow-up during the post-operative recovery period (12 weeks post-operatively) had significantly greater improvement in ROM up to 2 years after TSA compared to those with elevated pain during the post-operative recovery period, with multiple comparisons exceeding clinically relevant thresholds. Minimal pain during the post-operative recovery period was also associated with sustained lower pain levels compared to those with elevated pain up to 2 years from surgery.

Our study identified that patients with minimal pain at 12 weeks post-operatively sustained greater pre-operative to post-operative improvement of ROM (aTSA: abduction, FE, IR; rTSA: abduction, IR) up 2 years when compared to those with elevated pain. One possible explanation for these findings may be shoulder underutilization secondary to pain. We postulate that patients with elevated pain during the post-operative recovery period may limit shoulder activity and participate poorly in rehabilitation to avoid exacerbating symptoms. This behavioral avoidance could hinder ROM gains and promote stiffness, leading to poorer achievement of ROM. This is problematic because ROM is known to correlate with patient-perceived shoulder function, and a prior study demonstrated that improved ROM is associated with continued improvement in patient-reported outcome measures until 113° of abduction, 162° of FE, 52° of ER, and IR to L1.^12^ Our findings are also consistent with literature demonstrating that early post-operative rehabilitation and participation in functional exercises improve long-term outcomes when compared to those patients who delay rehabilitation and exercises.^5^ The results of this study are also consistent with current rehabilitation guidelines by the American Society of Shoulder and Elbow Therapists, which emphasize the restoration of passive ROM by 12 weeks post-operatively to optimize long-term outcomes.^3^^,^^16^ Patients who fail to achieve this milestone, possibly due to elevated pain, may fall behind in functional gains ultimately limiting their functional abilities. Notably, not all measured planes of ROM improved across groups. One possible explanation for this finding is that decreased pain may allow patients to independently engage earlier in common specific daily activities outside structured rehabilitation, such as personal hygiene, which would contribute to greater IR ROM.

The results of this study expand on previous work investigating the relationship of post-operative pain with long-term outcomes, most notably that of Judkins et al.^14^ In a retrospective review of patients undergoing aTSA or rTSA, the authors reported that patients with lower relative pain (defined as the lowest 50% of pain scores within the cohort) 24 hours post-operatively were correlated with improved American Shoulder and Elbow Surgeon scores, Shoulder Pain and Disability Index score, and Shoulder Pain and Disability Index 130 scores at 2 year follow-up, with the trend persisting even after controlling for changes in pain control and opioid use. While their focus pertained to the immediate post-operative window, our study extends this timeline and highlights pain level at 12 weeks post-operatively as a meaningful predictor. The authors in the aforementioned article proposed that the differences in outcomes were due to variations in an individual's pain sensitivity thresholds.^14^ In our study, we identified that patients with minimal pain at 12 weeks post-operatively had significantly improved pain on a daily basis and pain at worst up to 2 years post-operatively compared to those with elevated pain in aTSA and rTSA cohorts. Thus, patients with minimal pain in the post-operative period can expect better long-term pain levels as well. This raises the possibility that our findings of ROM differences between pain-level cohorts could also be explained, at least in part, by pain sensitivity thresholds mediating long-term function rather than a more efficacious analgesic regimen. For example, those with higher pain sensitivity thresholds may be able to move their shoulder across a greater degree of motion before cessation of the activity secondary to pain compared to those with low pain sensitivity thresholds at baseline. This aligns with work by Kadum et al,^15^ who found that patients with high pre-operative pain sensitivity thresholds had better Quick Disabilities of Arm, Shoulder and Hand scores at 12 months after undergoing stemless aTSA compared with those with low pre-operative pain sensitivity thresholds. However, it is not clear based on this study whether pain level is a modifiable risk factor for improving ROM post-operatively, and future prospective studies are necessary to elucidate whether pain control is causative to post-operative ROM or simply correlated.

A particularly interesting finding of our study is the sustained improvement in IR among patients with minimal pain, including the rTSA cohort, in which IR gains are often limited.^4^^,^^7^^,^^24^ Our results parallel those of Kim et al,^17^ who demonstrated that patients with lower IR scores had significantly worse pain at a mean follow-up of 18 months after rTSA compared to those with better IR scores. One possible explanation for this association may be related to coracoid or conjoint tendon impingement, which may function as both a generator of pain and mechanical constraint to IR.^18^ Kim et al^17^ further concluded that limited IR, perceived and measured, was an independent determining factor for lower ratings of patients' subjective level of success of their rTSA, while post-operative FE and ER had no correlation. Notably, we found no significant association between post-operative recovery pain and improvements in FE or ER in rTSA patients, suggesting that IR may be a more pain-sensitive marker of functional recovery.

These findings underscore the importance of achieving early pain control not only for short-term relief but also for long-term functional outcomes. If validated, 12-week pain assessments may serve as a clinical checkpoint to identify patients at risk for suboptimal long-term outcomes, and further prospective studies are needed to determine what modifiable factors–surgical, pharmacologic, behavioral, or rehabilitative intervention–can improve early pain control.

This study has several limitations. Although clinical outcomes in our TSA patients are evaluated prospectively, this study was conceived retrospectively. Our institution evaluates TSA patients at 2 weeks, 6 weeks, 12 weeks, 6 months, 1 year post-operatively, and annually thereafter. ROM and pain outcomes were not evaluated prior to the 6-week follow-up, limiting insights into the relationship between these 2 factors in the post-operative recovery period. While this is a limitation, the clinical relevance of marginally improved ROM within the first few weeks after TSA that does not persist to 6 weeks is unlikely to have clinical relevance to patients. In addition, duration of sling usage post-operatively was not recorded. Though rehabilitation protocols were standardized, immobilization ranged from 2 to 6 weeks. Longer periods of immobilization may limit early shoulder motion and delay improvement in ROM. While multiple surgeons contributed patients to this study, which enhances generalizability, differences in patient selection may have introduced bias and heterogeneity when selecting aTSA vs. rTSA; however, post-operative protocols were standardized across patients' surgeons for aTSA and rTSA. In addition, while we suggest that minimal pain level during the post-operative recovery period confers prognostic insights to long-term outcomes, we understand that elevated pain does not infer causation for inferior outcomes. As we did not evaluate strength, rotator cuff status, pre-operative opioid use, or intraoperative surgical characteristics, it is possible that patients with worse outcomes had predisposing factors leading to worse outcomes and increased pain levels. While these factors are not the focus of the study, they may have led to confounding of our results.

## Conclusion

Minimal pain during the post-operative recovery period at 12 weeks post-operatively was associated with sustained superior ROM and reduced pain up to 2 years following both aTSA and rTSA. These findings suggest that pain level during the post-operative recovery period may have important prognostic and potentially modifiable implications for long-term outcomes after TSA.

## Disclaimers:

Funding: No sources of funding.

Conflicts of interest: Dr Hao has a consultancy agreement with LinkBio Corp.

Dr Schoch is a paid consultant and receives royalties from Exactech, Innomed, and Responsive Arthroscopy.

Dr King is a paid consultant for Exactech, Inc. and LinkBio Corp.

Dr Thomas Wright is a paid consultant and receives royalties from Exactech, Inc.

Dr Farmer is a paid consultant for Exactech and Arthrex.

Dr Jonathan Wright is a paid consultant for Exactech, Inc.

Any additional authors, their immediate families, and any research foundations with which they are affiliated have not received any financial payments or other benefits from any commercial entity related to the subject of this article.
